# Pressor use and its impact on outcomes in aneurysmal subarachnoid hemorrhage patients with takotsubo cardiomyopathy: a quantitative analysis

**DOI:** 10.1007/s00701-026-06795-6

**Published:** 2026-02-17

**Authors:** Varun S. Shah, Paolo Lacanienta, Orlando Martinez, John Frances, Quang Nguyen, Adel Azghadi, Collin M. Labak, Christopher Michas, Zoey Knox, Will Casto, Alyssa Antuna, Xiaofei Zhou, Adam Bates, David L. Dornbos, Abhishek Ray, Yin C. Hu, Wei Xiong

**Affiliations:** 1 Department of Neurological Surgery, University Hospitals Cleveland Medical Center, Cleveland, OH USA; 2 Department of Neurology, University Hospitals Cleveland Medical Center, 11100, Euclid Avenue, Cleveland, OH 44106 USA; 3 Case Western Reserve University School of Medicine, Cleveland, OH USA; 4 Department of Neurosurgery, University of Kentucky, Lexington, KY USA; 5 Department of Emergency Medicine, University Hospitals Cleveland Medical Center, Cleveland, OH USA

**Keywords:** Aneurysm, Subarachnoid hemorrhage, Takotsubo cardiomyopathy, Vasopressor, Vasospasm

## Abstract

**Purpose:**

Takotsubo cardiomyopathy (TCM) is induced by catecholamine-induced cardiotoxicity. Though uncommon after aneurysmal subarachnoid hemorrhage (aSAH), TCM increases patient morbidity and mortality in an already-devastating disease process. The fear of worsening shock in TCM with blood pressure augmentation in patients with symptomatic vasospasm poses a dilemma for clinicians. This study aims to assess the relationship between vasopressor use after aSAH and TCM.

**Method:**

A multi-institutional chart review evaluated aSAH adult patients treated from 2015 to 2021. TCM diagnosis was the primary endpoint upon which the group was stratified. Baseline demographics, clinical presentation, aneurysm characteristics, adverse events, short-term outcomes, treatment variables, and vasopressor usage in average daily norepinephrine equivalent were collected.

**Results:**

311 patients were included; 13 (4.2%) had a TCM diagnosis. Median TCM diagnosis day was post-bleed day 4 (IQR 2–5). High-grade rupture (Hunt Hess score ≥ 3) was associated with TCM (*p* = 0.010). Vasopressor use after aneurysm securement was associated with TCM (*p* < 0.001). Vasospasm requiring multiple endovascular treatments, myocardial infarction, prolonged intubation, and general poor outcomes were significantly more prevalent in the TCM group on univariate analysis. On multivariate analysis, vasopressor use was not significantly associated with TCM (OR 4.275, CI 0.295–61.952, *p* = 0.287).

**Conclusion:**

In this sample of aSAH patients, a majority of TCM diagnoses were made within 5 days post-bleed. Most TCM patients were diagnosed prior to vasopressor initiation. Vasopressor use was not predictive of TCM in aSAH patients on multivariate analysis, suggesting its use is not a factor, though further study is needed.

## Introduction

Takotsubo cardiomyopathy (TCM) is an uncommon complication after aneurysmal subarachnoid hemorrhage (aSAH), affecting around 1–5% of this patient population [2, 6, 12, 13, 17, 18, 22]. Typical echocardiogram findings in TCM are decreased left ventricular ejection fraction with left ventricular midsegment dyskinesis, hypokinesis, or akineses with or without apical involvement. It is believed to be fueled by catecholamine-induced cardiotoxicity from physiologic stress [2, 9].

Though not causative factors, female sex, poor neurological presentation, large aneurysm size, and cerebral vasospasm are found to increase the odds of TCM in aSAH patients [2]. Additionally, a 2017 review on drug-induced cardiomyopathy identified 57 cases of induced TCM after exogenous catecholamine administration [7]. This form of cardiac dysfunction poses a clinical dilemma in aSAH patients needing pharmacologic systolic blood pressure augmentation to treat symptomatic cerebral vasospasm [7, 17]. TCM has been associated with worse outcomes in the aSAH population, showing the importance of identifying potentially modifiable risk factors to help mitigate its occurrence. The aims of this study were to characterize risk factors associated with the TCM in our multi-institutional patient sample of aSAH patients, with an emphasis on vasopressor use and its effects in this sample.

## Materials and methods

This retrospective study was approved by the Institutional Review Board (IRB STUDY20191604) at two academic tertiary-care institutions. Due to the retrospective nature of this study, the IRB deemed patient consent was not needed to conduct this study.

### Inclusion and exclusion criteria

A multi-institution, retrospective review evaluating aSAH patients aged 18 to 80 years old treated at the two institutions from 2015 to 2021 was undertaken. All aneurysm locations and treatment modalities were included. Exclusion criteria included pregnancy, non-aneurysmal or unknown etiology SAH, presence of prior aSAH, pediatric patients, presence of a mycotic aneurysm, presentation greater than 48 h after symptoms onset, or if the patient had care withdrawn prior to treatment.

### Primary and secondary outcomes

The primary outcome was development of TCM during the hospital stay. TCM was identified by echocardiographic diagnosis based off the attending cardiologist interpretation at both institutions. At both institutions, baseline echocardiograms were done within 24 h of admission after aSAH and then only again if there was clinical suspicion for heart failure (volume overload on clinical exam, increasing vasopressor requirement without blood pressure changes, decreasing cardiac index on monitoring). Secondary outcomes were hospital adverse events including myocardial infarction, pulmonary edema, deep vein thrombosis or pulmonary embolism, acute respiratory failure, need for hemicraniectomy for malignant edema, need for tracheostomy, need for gastrostomy (PEG), or need for shunt. Additional secondary outcomes included discharge disposition and modified Rankin score (mRS) at discharge and at 90 days.

Data collection points for univariate and multivariate analysis included demographic data, comorbidity status, Hunt Hess scale at presentation, modified Fisher Scale at presentation, vasopressor use as a categorical data point, norepinephrine (NE) equivalents used during hospitalization, development of symptomatic vasospasm (defined as a neurologic change with evidence of vasospasm on diagnostic cerebral angiogram), and number of endovascular vasospasm treatments.

### Vasopressor protocol

The typical MAP goal is MAPs > 65 with a systolic goal between 120–200 mmHg when there is no concern for vasospasm. Vasopressors are used in non-TCM patients when they are not meeting these goals and a cause for hypotension is investigated. When there is a concern for clinical vasospasm the SBP goal is 180–200, though if this is not possible due to cardiac demand concerns in patients with TCM the typical MAP goal is > 85.

### NE equivalents

NE equivalents were assessed by calculating the daily average dosage of vasopressor a patient was getting and then converting it to norepinephrine equivalent using the following formula [10]: norepinephrine dose (µg/kg/min) + epinephrine dose (µg/kg/min) + 1/10 × phenylephrine dose (µg/kg/min) + 1/100 × dopamine dose (µg/kg/min) + 1/8 × metaraminol dose (µg/kg/min) + 2.5 × vasopressin dose (U/min) + 10 × angiotensin II dose (µg/kg/min). This NE was then divided by the number of days the patient was on a pressor to get an average NE per day. Use of an inotrope was also recorded.

### Statistical analysis

Statistical calculations were performed using SPSS version 26 (IBM corp, Armonk, NY). The groups were separated by the presence of a TCM diagnosis versus no TCM diagnosis. Prior to running statistical tests, the distribution of each variable was assessed. Those with a normal distribution had parametric analyses and distributions with a skew were analyzed using non-parametric tests. Pearson chi square and Student’s t-test were used to assess categorical and continuous variables, respectively. Mann–Whitney U test was undertaken to assess ordinal variables. Outcomes were assessed with univariate and multivariate logistic regression analysis. A Bonferroni correction was applied to account for multiple comparisons when conducting sequential univariate analyses. An alpha of 0.05 was set to determine significance.

## Results

### Demographic data

After exclusion, a total of 311 patients were included in the final analysis. TCM affected 4.2% (*n* = 13) of the cohort. Baseline demographics between the two groups can be found in Table 1. The average age of the TCM group was 59.8 ± 14.1 years, which was similar to the non TCM group at 56.4 ± 14.0 years (*p* = 0.906). The majority of the TCM group was female (92.3%) compared to 66.8% of the non-TCM group, though this result was not statistically significant (*p* = 0.054). Pre-existing coronary artery disease (CAD), hypertension (HTN), or hyperlipidemia (HLD) was not found to be significant between groups. Only 2 patients in the TCM group had pre-existing CAD, none of the patients in the TCM group had pre-existing heart failure. Tobacco use was more prevalent in the non-TCM group (50% vs 15.4%, *p* = 0.015). Additionally, the median day of TCM diagnosis was post-bleed day 4 (IQR 2, 5). No TCM patient required mechanical circulatory assist devices.

**Table 1:** Baseline demographics

	TCM (*n* = 13)	No TCM (*n* = 298)	*p*-value
Age (years)	59.8 ± 14.1	56.4 ± 14.0	0.906
Female	92.3% (12)	66.8% (199)	0.054
Caucasian	76.9% (10)	75.5% (225)	0.907
African American	2	61	0.655
Other	1	10	0.407
CHF	0	5	0.638
HTN	10	177	0.206
HLD	5	53	0.061
T2DM	0	33	0.204
COPD	2	25	0.381
ESRD	0	2	0.767
CAD	2	18	0.179
Connective tissue disorder	0	10	0.502
Afib	1	14	0.622
VTE	0	8	0.550
Liver disease	0	3	0.716
Bleeding dyscrasia	0	4	0.674
AC/AP use	3	73	0.907
Smoking	2	149	0.015
Illicit use	0	34	0.188
Etoh abuse	1	33	0.702
uninsured	0	27	0.256
***Avg day dx w/TCM***	***4 (2, 5)***	***-***	

*AC/AP* anticoagulants/antiplatelets, *CAD* coronary artery disease, *ESRD* end stage renal disease, *HLD* hyperlipidemia, *HTN* hypertension, *sCHF* symptomatic congestive heart failure, *T2DM* type 2 diabetes mellitus, *TCM* Takotsubo cardiomyopathy, *VTE* venous thromboembolism

Clinical characteristics between groups can be found in Table 2. The majority of the TCM group (76.9%) was high-grade rupture (defined at a Hunt-Hess grade 3 or higher), compared to 40% of the non-TCM group having high-grade ruptures (*p* = 0.010). All the TCM group were a modified Fischer (mF) grade 3 or 4, as was majority of the non-TCM group (81.2%) (*p* = 0.106). Aneurysm location, need for external ventricular drain (EVD), method of treatment and aneurysm side were not statistically significant between groups. Pressor usage was statistically more prevalent in the TCM group (76.9% v 30.5%, *p* < 0.001), though the median NE per day (*p* = 0.768), total NE (*p* = 0.366), days on pressors (*p* = 0.054), and number of pressors (*p* = 0.051) was not significantly different between groups. One patient in the TCM group required vasopressors for septic shock, and another for cardiogenic shock, but the rest required vasopressors for vasospasm. All of the non-TCM group was on vasopressors to augment BP for treatment of clinical vasospasm. The most common vasopressor used was norepinephrine, second line was vasopressin, and third line was phenylephrine. Inotropes were added if further cardiac augmentation was needed. Of all the patients requiring vasopressors 17% were on 1 pressor, 58.5% were on 2 pressors, and 24% were on 3 pressors. Norepinephrine was used in 98.1% of patients (there was 1 patient on inotropes only), vasopressin was used in 58.5% of patients, phenylephrine was used in 24.5% of patients, dobutamine was used in 7.5% of patients, and milrinone was used in 15.1% of patients.

**Table 2:** Clinical characteristics

	TCM (*n* = 13)	No TCM (*n* = 298)	*p*-value
***HH 3 or higher***	***10***	***119***	***0.010***
Modified Fischer 3 or 4	13	242	0.106
Infratentorial aneurysm	5	54	0.067
EVD on arrival	10	150	0.071
rebleed	0	6	0.595
Clipped aneurysm	5	102	0.753
Coiled aneurysm	8	197	0.753
Left side aneurysm	3	10	0.460
***Vasopressor use***	***10***	***91***	** < *0.001***
NE equiv per day	0.289 (0.017, 0.427)	0.278 (0.094, 0.391)	0.768
Total number of pressors	3 (IQR 1, 3)	2 (IQR 1, 2)	0.051
Days on pressors	5.4 ± 3.4 days	8.3 ± 3.8 days	0.054

*EVD* external ventricular drain, *HH* Hunt Hess scale, *NE* norepinephrine, *SAH* subarachnoid hemorrhage, *TCM* Takotsubo cardiomyopathy

### Adverse events and TCM

Adverse events and short-term outcomes characteristics can be found in Tables 3 and 4. In hospital mortality rates were not significantly different between groups (7.7% in TCM group, 8.4% in the non-TCM group, *p* = 0.93). There was no statistically significant difference in vasospasm rates between groups, though of those who developed clinical vasospasm 75% (6/8) of the TCM group needed multiple endovascular interventions for vasospasm compared to 39.0% (46/118) of the non-TCM group (*p* = 0.045). Myocardial infarction (MI) was more prevalent in the TCM group (*p* < 0.001) as was mechanical ventilation (*p* = 0.003), need for tracheostomy (*p* = 0.007), and need for PEG placement (*p* = 0.009). None of the myocardial infarction patients needed cardiac catheterization. All of these patients developed had NSTEMI due to demand ischemia from vasopressor use. Of the two MI patients in the TCM group, one was found to have an NSTEMI and significant troponemia while on vasopressors for blood pressure augmentation, and on their echocardiogram they were diagnosed with TCM. The other TCM patient was found to have an NSTEMI on workup of significant troponemia and chest pain which initiated an EKG, this was after their TCM diagnosis. A good disposition (defined as discharge to home or acute rehab) was more prevalent in the non-TCM group (38.5% vs 61.7% *p* = 0.005).

**Table 3:** Adverse events

	TCM (*n* = 13)	No TCM (*n* = 298)	*p*-value
Vasospasm	8	118	0.120
***Multiple vasospasm tx***	***6***	***46***	***0.045***
***Myocardial infarction***	***2***	***1***	** < *0.001***
Pulmonary edema	1	6	0.185
VTE	1	11	0.478
***Intubation***	***10***	***104***	***0.003***
Hemicraniectomy for edema	1	14	0.651
***Tracheostomy***	***5***	***37***	***0.007***
***PEG***	***7***	***67***	***0.009***
Shunt	3	37	0.261
In hospital mortality	1 (7.7%)	25 (8.4%)	0.930

*PEG* gastrostomy, *TCM* Takotsubo cardiomyopathy, *VTE* Venous thromboembolism

**Table 4:** Short-term outcome characteristics

	TCM (*n* = 13)	No TCM (*n* = 298)	*p*-value
***Home/rehabilitation disposition***	***5***	***184***	***0.005***
Good functional status (mRS 0–3)	4	169	0.178

*mRS* modified Rankin score, *TCM* Takotsubo cardiomyopathy

### Vasospasm and TCM

On univariate analysis, TCM was not associated with increased odds of vasospasm (odds ratio [OR] 2.414, 95% confidence interval [CI] 0.771–7.557, *p* = 0.130). Additionally, vasospasm was not associated with increased odds of TCM diagnosis (OR 2.413, *p* = 0.130). Additionally, the association between TCM diagnosis and the need for multiple endovascular treatments for vasospasm lost statistical significance (OR 4.696, 95% CI 0.909–24.268, *p* = 0.065). TCM increased the odds of in-hospital MI (OR 53.273, 95% CI 4.485–632.83, *p* = 0.002). Acute respiratory failure, need for tracheostomy, and need for PEG also increased in patients with TCM, though on multivariate analysis all three of these variables lost significance (Tables 5 and 6). On multivariate analysis, MI was the only outcome that remained significant (OR 43.817, 95% CI 3.513–546.57, *p* = 0.003). Patients with a TCM diagnosis had decreased odds of a good disposition (OR 0.217, 95% CI 0.069–0.689, *p* = 0.009). On univariate analysis, vasopressor use increased odds of TCM diagnosis (OR 7.582, 95% CI 2.039–28.202, *p* = 0.003), though on multivariate analysis pressor use was not an independent predictor of TCM in aSAH patients (OR 4.275, 95% CI 0.295–61.952, *p* = 0.287).

**Table 5:** Impact of TCM dx on adverse events and outcomes (univariate analysis)

	OR	95% CI	*p*-value
Vasospasm	2.414	0.771–7.557	0.130
Multiple endovascular treatments for vasospasm	4.696	0.909–24.268	0.065
***MI***	***53.273***	***4.485–632.83***	***0.002***
Pulmonary edema	3.958	0.441–35.524	0.219
VTE	2.121	0.253–17.798	0.488
Intubation	5.962	1.605–22.146	0.008
Hemicraniectomy for edema	1.619	0.196–13.348	0.654
***Trach***	***4.409***	***1.369–14.194***	***0.013***
***PEG***	***4.022***	***1.307–12.375***	***0.015***
Shunt	2.116	0.557–8.044	0.271
***Home/rehabilitation facility disposition***	***0.217***	***0.069–0.689***	***0.009***
Good functional status (mRS 0–3)	0.391	0.095–1.607	0.193

*CI* confidence interval, *MI* myocardial infarction, *mRS* modified Rankin score, *OR* odds ratio, *PEG* gastrostomy, *TCM* Takotsubo cardiomyopathy, *VTE* Venous Thromboembolism

**Table 6:** Impact of TCM dx on adverse events and outcomes (multivariate analysis)

	OR	95% CI	*p*-value
**MI**	**43.817**	**3.513–546.57**	**0.003**
Intubation	0.744	0.143–3.878	0.726
Tracheostomy	3.226	0.497–20.937	0.220
PEG	0.821	0.087–7.745	0.863

*CI* confidence interval, *MI* myocardial infarction, *OR* odds ratio, *PEG* gastrostomy, *TCM* Takotsubo cardiomyopathy

## Discussion

### Key results

In this small population, a majority of aSAH patients were diagnosed with TCM within 5 days of subarachnoid hemorrhage. Most patients were diagnosed prior to initiating vasopressors and vasopressor use was not predictive of subsequent development of TCM in this population.

### Interpretation

Unsurprisingly, worse outcomes were more prevalent in the TCM group, which is consistent with existing literature. A single institution study published a 77% rate of poor outcome in their TCM group compared to 47% in their non-TCM aSAH group [2], though this group defined mRS > 2 as a poor outcome. Another study saw in their cohort of 26 TCM patients, only 27% had poor outcome (defined as an mRS > 3 at last follow up in the cited study [17]. Another study by Kilbourn et al. found their TCM group to be statistically more likely to suffer a worse outcome [8]. The study by Inamasu et al. found that 43% of their group had a 90-day mRS < 3, though no comparison was made to a non-TCM group [5]. Although our study was not able to report long-term outcomes, the prevalence of tracheostomy, PEG placement, and lower rates of discharge to home or rehabilitation facilities do suggest long-term outcomes are worse in our TCM group compared to our non-TCM group.

On short-term outcome assessment, Abd et al. found a 26% in-hospital mortality rate in their TCM cohort, but again no comparison was made to a non-TCM group [1]. Overall, the reported in-hospital mortality for TCM patients in the 6 published case series range from 20% to 36.4% [1, 2, 4, 5, 8, 17], which contrasts with 7.7% of our TCM cohort suffering an in-hospital mortality (compared to 11.9% of the non-TCM cohort). The literature suggests prognosis in TCM patients with aSAH is primarily driven by the severity of aSAH [5, 14, 21]. Long-term studies of TCM patients show an overall-mortality of 5.6% per patient-years [19], which is lower than the reported in-hospital mortality rate of 10–20% for aSAH, and lower than the long-term mortality for aSAH as well [2, 11, 16]. Additionally, another study by Norberg et al. highlighted most death in SAH patients within 3 months of aneurysm rupture are neurologic in nature, and rarely cardiac [15]. The current study including all other published cohorts identify worse neurologic status on presentation to be predictive of TCM, though these worse neurologic statuses are also predictive of worse outcomes. This is not meant to suggest ignoring cardiac function for the sake of brain perfusion as these short-term cardiac complications worsen prognosis [4, 21].

Consistent with other studies, vasospasm was more prevalent in our TCM group though this was not statistically significant nor was TCM predictive of vasospasm [2]. Many studies have shown TCM is an early diagnosis in aSAH patients. In this current study, the median day of TCM diagnosis was post-bleed day 4, and most patients were diagnosed post-bleed day 5 or earlier. Another study diagnosed their patients with TCM with their initial echocardiogram, which was obtained on average post-bleed day 2 [1]. This finding was reflected in the prospective cohort study by Kilbourn et al., finding all their patients were diagnosed with TCM on their initial echocardiogram within 48 h of aneurysm rupture [17]. The study by Talahma et al. found their cohort was diagnosed with TCM on post-bleed day 1 to 10, with an average diagnosis on post-bleed day 5 [17]. In this study, those with an earlier diagnosis had a tendency towards poor outcomes, which is also reflected in the results of the Barrow study whose TCM patients with good outcome were diagnosed on average on post-bleed day 6 and those with a negative outcome were diagnosed on average post-bleed day 5 [2, 17]. Given the timing of TCM diagnosis, this suggests the cardiac dysfunction is likely not preventable, furthermore it suggests the treatment of vasospasm does not contribute to the development of TCM [8, 17]. In the Barrow study, roughly half of their TCM group with negative outcome underwent endovascular therapy for vasospasm, compared to 100% of their group with good outcome [2]. This suggests that aggressive endovascular treatment of vasospasm in TCM patients may lead to improved outcomes.

It is currently unknown what predicts a poor response to intraarterial calcium channel blockers for cerebral vasospasm treatment. 75% (6/8) of the TCM population in our study that underwent vasospasm treatments required multiple endovascular interventions for vasospasm compared to 39% of the non-TCM group. 4/6 of these patients were high grade ruptures and 5/6 had a mF4 grade scan. 4/6 aneurysms were in the supratentorial space. 2/6 patients had intraarterial balloon angioplasty and another 2/6 patients had intrathecal nicardipine. Intraarterial calcium channel blockers have vasodilatory effects, infusion into the internal carotid artery typically cause local vasodilatory effects though systemic effects can arise. A hypothesis is that TCM patients may not be able to tolerate the effects of intraarterial CCB given sensitivity to its effects. There is a case report of cardiac arrest after intraarterial verapamil for vasospasm treatment that could show the hypersensitivity these patients have to CCB [20]. There is no large body of literature on this though this lends itself to an area of study.

Hyperdynamic therapy and hypervolemia are the typical first line therapies for patients with clinical vasospasm, though the diminished cardiac output found in TCM limit the hypertensive effects of vasopressors and can worsen cardiac dysfunction [2, 3]. Pressor use was more prevalent in the TCM population in this current study, which is consistent with the prospective cohort in the Kilbourn study [8]. In our study, pressor use was not predictive of TCM, and surprisingly the average NE, total NE, and days on pressors was not significantly different between TCM patients and non TCM patients. Most of the TCM patients in our cohort (10 out of 13) were diagnosed with TCM prior to any vasopressor need arising. Given the hypothesis that TCM is catecholamine related and the high percentage of patients in our cohorts that received vasopressors for vasospasm, we might have expected a rise TCM diagnoses later in the hospital course. We did not observe this pattern in our population. Multiple studies have found no significant difference in vasopressor use and the incidence of TCM. The Barrow study found days on pressors and number of pressors were similar between their TCM patients with good outcomes and bad outcomes [2]. Pressor use was not predictive of outcomes in the Kibourn et al. study, and the conclusion was made that pressor use did not contribute to TCM development [8]. Additionally, the Talahma et al. study found the use of vasopressors prior to TCM diagnosis and continuing them after diagnosis was not associated with poor outcome, and trended toward a good outcome, suggesting vasospasm treatment in aSAH patients is associated with improved outcomes [17]. The early occurrence of TCM after aSAH in our study and the literature suggests acute medical illness and treatment of vasospasm with pressors does not predispose to TCM development, and further suggests treating optimizing brain perfusion results in the best outcomes of aSAH regardless of TCM.

### Limitations

Limitations of this study are those inherent to retrospective review. There were patients who had incomplete datasets and needed to be excluded. Additionally, more than 60% of patients did not have accurate follow-up data given recent changes in the hospital’s electronic medical record, therefore long-term neurologic and cardiac outcomes could not be analyzed. This electronic medical record change also impacted the ability to obtain results on delayed cerebral ischemia, further impacting generalizability and long-term result interpretation. This is a cohort of largely Caucasian patients from the Midwest, which could potentially impact results. Given this was a retrospective review, no a priori power analysis was performed. This unfortunately reflected in statistical analysis as some variables were approaching significance the study is likely underpowered to reach significance. The comparison of the small number of TCM patients to the rest of the sample account for the large confidence intervals on statistical analysis. Unfortunately, this could not be accounted for given TCM is a rare diagnosis, and this is a retrospective review, though the purpose of this paper is to add to the existing body of literature on TCM in aSAH. TCM is a rare, though morbid, complication of aSAH; to obtain meaningful results, large multicenter studies are necessary to obtain an adequate sample size. Given this is a multicenter review, outcomes could be affected by differences in management though initial analysis comparing the individual cohorts showed no significant differences in baseline or outcomes between groups. There is a potential for selection bias in echocardiogram ordering patterns across institutions, though both institutions obtain echocardiogram within 24 h of admission after aSAH and then again during the hospital stay only if there is a clinical suspicion for cardiac abnormality. Institutional differences in vasopressor protocols and vasospasm treatment protocols may also impact results, as this could impact if inotropes are used at a certain point which could affect the NE equivalents a patient may have. Additionally, if a practitioner is more aggressive with angiographic treatment for vasospasm over others this could also affect results. There are multiple definitions of TCM in the literature; if another definition were used, the cohort of TCM patients may have been different and could have affected results. In this study, TCM was diagnosed exclusively from echocardiogram. EKG findings were not analyzed or used as part of the diagnostic criteria as they were non-specific, which could have impacted diagnosis numbers and associated results.

## Conclusion

TCM is a rare but morbid complication of aSAH, and its pathophysiology is largely unknown. This is the first study to quantitatively analyze vasopressor use, and in this population, it is not predictive of a TCM diagnosis. Large scale, multicenter, studies are needed to comment on the safety of vasopressor use in the aSAH population with TCM.

## Data Availability

No datasets were generated or analysed during the current study.
